# Gray Matter and White Matter Abnormalities in Temporal Lobe Epilepsy Patients with and without Hippocampal Sclerosis

**DOI:** 10.3389/fneur.2018.00107

**Published:** 2018-03-13

**Authors:** Iman Beheshti, Daichi Sone, Farnaz Farokhian, Norihide Maikusa, Hiroshi Matsuda

**Affiliations:** ^1^Integrative Brain Imaging Center, National Center of Neurology and Psychiatry, Kodaira, Japan; ^2^College of Life Science and Bioengineering, Beijing University of Technology, Beijing, China

**Keywords:** temporal lobe epilepsy, hippocampal sclerosis, voxel-based morphometry, FreeSurfer, magnetic resonance imaging

## Abstract

The presentation and distribution of gray matter (GM) and white matter (WM) abnormalities in temporal lobe epilepsy (TLE) have been widely studied. Here, we investigated the GM and WM abnormalities in TLE patients with and without hippocampal sclerosis (HS) in five groups of participants: healthy controls (HCs) (*n* = 28), right TLE patients with HS (*n* = 26), right TLE patients without HS (*n* = 30), left TLE patients with HS (*n* = 25), and left TLE patients without HS (*n* = 27). We performed a flexible factorial statistical test in a whole-brain voxel-based morphometry analysis to identify significant GM and WM abnormalities and analysis of variance of hippocampal and amygdala regions among the five groups using the FreeSurfer procedure. Furthermore, we conducted multiple regression analysis to assess regional GM and WM changes with disease duration. We observed significant ipsilateral mesiotemporal GM and WM volume reductions in TLE patients with HS compared with HCs. We also observed a slight GM amygdala swelling in right TLE patients without HS. The regression analysis revealed significant negative GM and WM changes with disease duration specifically in left TLE patients with HS. The observed GM and WM abnormalities may contribute to our understanding of the root of epilepsy mechanisms.

## Introduction

Temporal lobe epilepsy (TLE) is a common form of epilepsy that originates in the temporal lobes and is usually characterized by unpredictable seizures. Hippocampal sclerosis (HS), as one of the most common pathological findings in TLE, is identified by neuronal cell loss in some hippocampal regions, such as the cornu ammonis 1 area (CA1) (1). TLE patients with HS (TLE-HS) account for about 65% of TLE patients and suffer from significant neuronal cell loss and gliosis in hippocampal regions (2). Individuals with TLE-HS experience focal seizures with impaired awareness. The main factors in the development of HS are febrile seizures and inflammatory and genetic susceptibility (1). TLE patients without an abnormal brain structure (TLE-no) account for about 35% of all TLE patients. These individuals have very mild or absent neuronal atrophy in hippocampal regions (2).

In TLE patients with and without HS, structural damages are not limited to the temporal lobe, but also extend to other regions. For example, several researchers reported significant structural abnormalities in the entorhinal cortex, parahippocampal and fusiform gyrus, thalamus, basal ganglia, and frontal and parietal lobe regions in TLE-HS patients (3, 4), whereas the structural abnormalities in TLE-no patients tend to be more unpretentious (3, 5).

Various studies have investigated the brain abnormalities in TLE in scenarios such as white matter (WM) abnormalities in TLE patients with amygdala enlargement (AE) (6), gray matter (GM) and WM abnormalities in TLE patients with and without HS (7), side matters in TLE-HS and TLE-no patients (8), and WM changes in medial TLE (9–12). Despite the recent research into epilepsy, its causes remain unclear, and further studies of its progression are needed.

In the present study, we aimed to investigate the presentation and distribution of brain morphological abnormalities in TLE patients with and without HS in comparison with healthy controls (HCs) as well as a volumetric analysis on hippocampal and amygdala volumes. Besides, another objective of this study was to explore the brain morphological changes with duration of disease as a clinical feature among TLE patient groups. With respect to these points, we investigated the GM and WM abnormalities in TLE at three levels. At the first level, we used a flexible factorial statistical test *via* whole-brain voxel-based morphometry (VBM) analysis to identify significant GM and WM abnormalities among five groups of participants: HCs and patients with either right TLE with HS (RTLE-HS), right TLE with no HS (RTLE-no), left TLE with HS (LTLE-HS), or right TLE with no HS (RTLE-no). Several recent studies have used the VBM procedure to detect the brain morphological alterations in various brain diseases, such as Alzheimer’s disease (13, 14), Parkinson’s disease (15), epilepsy (16), and schizophrenia (17, 18). At the second level, we conducted a FreeSurfer analysis to investigate hippocampal and amygdala volumes as the main regions affected by epilepsy in the VBM analysis. At the third level, we investigated the regions of GM and WM changes with an increasing duration of disease through multiple regression analysis in Statistical Parametric Mapping software (SPM12).

## Subjects and Methods

### Subjects

All subjects were native Japanese speakers. The data used in this study were acquired at the National Center of Neurology and Psychiatry Hospital, Tokyo, Japan, between November 2013 and January 2017. The magnetic resonance imaging (MRI) images were all T1-weighted structural MRI results from 3T scanners (repetition time = 7.12 ms, echo time = 3.4 ms, flip angle = 10°, matrix = 260 cm × 320 cm, field of view = 26 cm × 24 cm, acquisition time = 4.01 min, voxel size of 0.81 mm × 0.81 mm × 0.6 mm) manufactured by Philips and using the Digital Imaging and Communications in Medicine (DICOM) format. A total of 136 participants were recruited and classified into five groups: 28 HCs (16 men and 12 women, 40.67 ± 10.97 years of age), 26 RTLE-HS patients (12 men and 14 women, 42.07 ± 11.53 years of age), 30 RTLE-no patients (16 men and 14 women, 43.76 ± 13.78 years of age), 25 LTLE-HS patients (8 men and 17 women, 38.00 ± 13.11 years of age), and 27 RTLE-no patients (13 men and 14 women, 39.14 ± 13.12 years of age). The patients with an HS or non-HS diagnosis were assessed by visual inspection of the MRI findings; the patients with an HS diagnosis were recognized as having different criteria: ipsilateral reduced hippocampal volume, increased T2 signal at the hippocampus, and abnormal morphology (i.e., a loss of internal architecture of the stratum radiatum, a thin layer of WM separating the dentate nucleus and Ammon’s horn). Among the 108 TLE patients, 98 were drug resistant, with the remainder (10 TLE patients) drug responsive. In addition, 21 patients underwent ictal EEG recording as well as other presurgical examinations, whereas diagnoses for the others patients were conducted based on clinical semiology and interictal epileptiform discharges. More details on the participants’ demographic and clinical characteristics are provided in Table 1. All participants gave written informed consent. The study was approved by the Institutional Review Board at the National Center of Neurology and Psychiatry Hospital.

**Table 1:** Participants’ clinical characteristics.

	HC	RTLE-HS	RTLE-no	LTLE-HS	LTLE-no
*n*	28	26	30	25	27
Age, years (range)	40.67 ± 10.97 (20–62)	42.07 ± 11.53 (20–61)	43.76 ± 13.78 (20–68)	38.00 ± 13.11 (17–64)	39.14 ± 13.12 (18–65)
Onset age, years (range)	– –	15.80 ± 13.24 (1–47)	28.80 ± 18.71 (3–62)	12.04 ± 8.26 (2–28)	22.47 ± 14.98 (2–64)
Duration disease, years (range)	– –	26.26 ± 11.18 (7–47)	14.96 ± 13.65 (0–43)	25.96 ± 11.46 (1–46)	16.40 ± 15.97 (0–53)
Female/male	12/16	14/12	14/16	7/8	14/13
Drug-resistant patients	–	25	25	24	24

*HC, healthy control; TLE, temporal lobe epilepsy; HS, hippocampal sclerosis; R, right; L, left; RTLE-HS, right TLE with HS*.

### MRI Preprocessing

The raw DICOM scans were reviewed and converted to NIfTI (Neuroimaging Informatics Technology Initiative) format using MRICRON software.^1^ At the first level, all MRI scans were corrected for bias field inhomogeneities and then segmented into GM, WM, and cerebrospinal fluid components through the VBM technique implemented in the CAT toolbox^2^ in the software program SPM12.^3^ We used the Diffeomorphic Anatomic Registration Through Exponentiated Lie (DARTEL) algebra algorithm to normalize the segmented scans into standard Montreal Neurological Institute space. The DARTEL approach helps to obtain precise and accurate localization of structural damage on MRI images (19). The segmented and normalized scans were modulated using a nonlinear deformation.

Herein, we used GM and WM components to identify the abnormalities in TLE patients with and without HS. Finally, we used an 8-mm full-width-half-maximum Gaussian smoothing kernel to smooth all of the GM and WM components. In addition to revealing the GM and WM abnormities, we investigated the hippocampal and amygdala volumes in the five groups of participants (i.e., HC, RTLE-HS, RTLE-no, LTLE-HS, and LTLE-no). Thus, at the second level, we performed a FreeSurfer analysis to extract the respective volumes from three-dimensional T1-weighted MRI scans of all of the subjects. We used the FreeSurfer program ver. 5.3.0.^4^ The technical details of the FreeSurfer analysis were as described (20–22). The hippocampal and amygdala volumes were adjusted for estimated total intracranial volume (eTIV) and age as follows (23):

(1) $$\begin{array}{cc} {\text{Volume}}_{\text{adjusted subject}}\text{=} & {\text{Volume}}_{\text{raw subject}}-\beta_{1}\left({\text{eTIV}}_{\text{subject}}-{\text{eTIV}}_{\text{mean}}\right)\\ & -\beta_{2}\left({\text{Age}}_{\text{subject}}-{\text{Age}}_{\text{mean}}\right) \end{array}$$

where β_1_ and β_2_ are the slopes of the linear regression lines between the eTIV and the volume of interest and between the age and the volume of interest, respectively, in the HC group. Besides, the eTIV_mean_ and the Age_mean_ stand the means of eTIV and age for all HCs, respectively.

### Statistical Analysis

After the spatial preprocessing, the GM and WM images were subjected to a flexible factorial analysis in SPM12. A family-wise error with a *p*-value of less than 0.05 was used for group comparisons, and the extent threshold was set at 50 voxels. Regional alterations in GM and WM volumes were detected by a voxel-based analysis of the entire brain. To investigate the hippocampal and amygdala volumes obtained from the FreeSurfer analysis, we performed an analysis of variance (ANOVA) followed by a Tukey multiple comparison with *p* < 0.05 implemented in Statistical Package for Social Sciences software ver. 16.0 (IBM, Armonk, NY, USA) as well as a *Z* score analysis using the following formula:

(2) $$Z=\frac{x-\mu }{\sigma },$$

where *x* stands for individual adjusted volume, μ and σ are the mean and SD of respective adjusted volumes in the HC group. Furthermore, we investigated the GM and WM changes with the duration of disease through a multiple regression analysis in SPM12 using an uncorrected threshold of *p* < 0.001. In the regression analysis, the extent threshold was set at 250 voxels. For all VBM analyses (namely, the flexible factorial statistical test and regression analysis), the subject’s age, sex, and eTIV were considered in the matrix design in the SPM12.

## Results

### VBM of the Subjects’ GM

**Figure 1:**
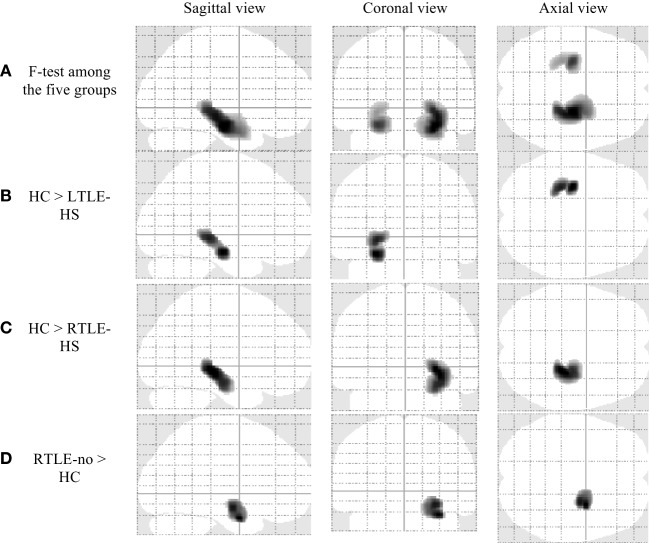
Significant gray matter volume alterations in the five groups by voxel-based morphometry analysis using SPM12 (family-wise error-corrected at *p* < 0.05 and an extent threshold *K* of 50). **(A)** F-test among the five groups, **(B)** HC > LTLE-HS, **(C)** HC > RTLE-HS, and **(D)** RTLE-no > HC. HC, healthy control; LTLE-HS, left temporal lobe epilepsy with hippocampal sclerosis; RTLE-HS, right temporal lobe epilepsy with hippocampal sclerosis; RTLE-no, right temporal lobe epilepsy without hippocampal sclerosis.

Figure 1 and Table 2 show significant GM volume alterations in our whole-brain VBM analysis among the five groups. Compared with the HCs, the LTLE-HS and RTLE-HS subjects showed a significant GM reduction in the left and right hippocampal regions, respectively (Figures 1B,C). The VBM analysis also revealed a significant increase in the GM in the right amygdala in the RTLE-no subjects compared with the HCs (Figure 1D). The VBM analysis showed no significant GM volume alterations in the LTLE-no group compared with the HCs or in the reverse contrast.

**Table 2:** Clusters of gray matter alterations shown by the voxel-based morphometry analysis using statistical parametric mapping software.

Analysis	Location of peak voxels	Hemisphere	Cluster size (no. of voxels)	Talairach coordinates (*x*,*y*,*z*)	MNI coordinates (*x*,*y*,*z*)	*Z*-value (peak voxel)
(a) *F*-test among the five groups	Hippocampus Hippocampus	R L	4,996 1,820	26, −16, −13 −26, −17, −11	27, −16, −20 −27, −16, −18	Inf Inf
(b) HC > LTLE-HS	Hippocampus	L	1,281	−26, −18, −11	−27, −16, −18	7.93
(c) HC > RTLE-HS	Hippocampus	R	2,201	27, −28, −2	28, −27, −8	10.36
(d) RTLE-no > HC	Amygdala	R	1,256	29, −5, −19	30, −2, −27	4.8

*Anatomical regions were derived from the Talairach Client program*.

*L, left hemisphere; R, right hemisphere; MNI, Montreal Neurological Institute (family-wise error-corrected at *p* < 0.05); RTLE-HS, right TLE with HS; LTLE-HS, left TLE with HS*.

### VBM of WM Analysis

**Figure 2:**
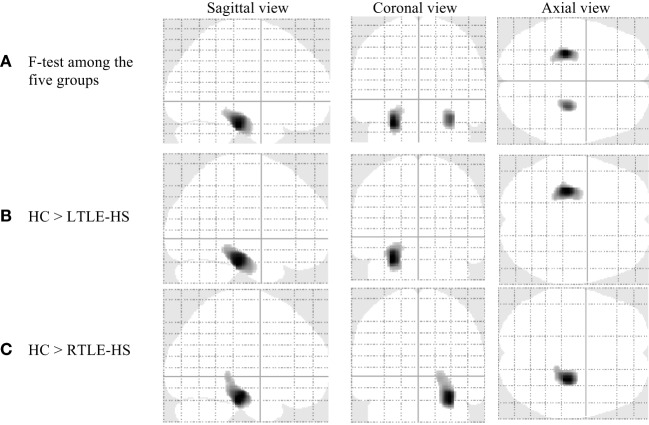
Significant white matter volume alterations in the five subject groups shown by the voxel-based morphometry analysis using SPM12 (family-wise error-corrected at *p* < 0.05 and an extent threshold *K* of 50). **(A)** F-test among the five groups, **(B)** HC > LTLE-HS, and **(C)** HC > RTLE-HS. HC, healthy control; LTLE-HS, left temporal lobe epilepsy with hippocampal sclerosis; RTLE-HS, right temporal lobe epilepsy with hippocampal sclerosis.

Significant WM volume alterations based on the whole-brain VBM analysis in the five groups are shown in Figure 2 and Table 3. Compared with the HCs, a significant WM reduction was observed in the left and right parahippocampal regions in the LTLE-HS and RTLE-HS patients, respectively (Figures 2B,C). The WM reductions were observed in adjacent areas of GM in medial temporal lobes. There were no significant WM volume alterations in the LTLE-no and RTLE-no groups compared with the HCs or in the reverse contrast.

**Table 3:** Clusters of white matter alterations by the voxel-based morphometry analysis using statistical parametric mapping software.

Analysis	Location of peak voxels	Hemisphere	Cluster size (no. of voxels)	Talairach coordinates (*x*,*y*,*z*)	MNI coordinates (*x*,*y*,*z*)	*Z*-value (peak voxel)
(a) *F*-test among the five groups	Parahippocampal Parahippocampal	L R	1,043 632	−25, −27, −18 28, −23, −14	−27, −26, −26 30, −22, −21	7.25 6.51
(b) HC > LTLE-HS	Parahippocampal	L	1,376	−26, −28, −17	−28, −27, −24	8.10
(c) HC > RTLE-HS	Parahippocampal	R	1,291	27, −23, −14	28, −22, −21	7.98

*Anatomical regions were derived from the Talairach Client program*.

*L, left hemisphere; R, right hemisphere; MNI, Montreal Neurological Institute (family-wise error-corrected at *p* < 0.05); RTLE-HS, right TLE with HS; LTLE-HS, left TLE with HS*.

### Hippocampal and Amygdala Volumes by FreeSurfer

**Figure 3:**
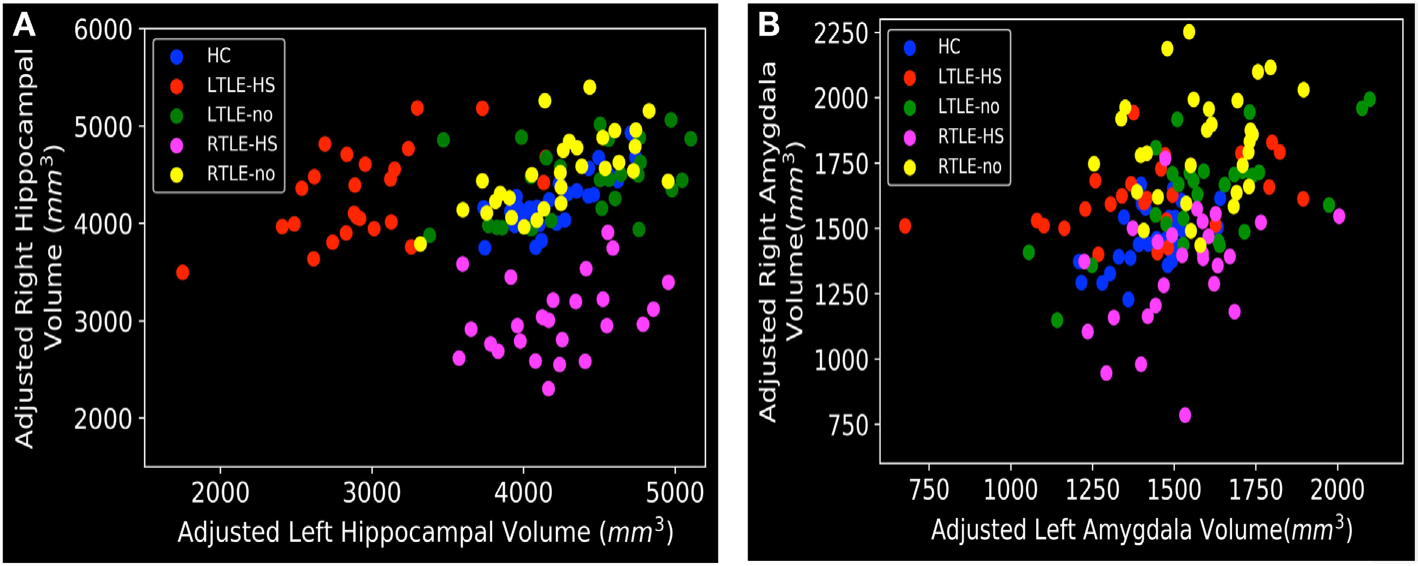
Distribution of left and right volumes among the five subject groups, obtained from the FreeSurfer results: **(A)** hippocampal and **(B)** amygdala. HC, healthy control; LTLE-HS, left temporal lobe epilepsy with hippocampal sclerosis; LTLE-no, left temporal lobe epilepsy without hippocampal sclerosis; RTLE-HS, right temporal lobe epilepsy with hippocampal sclerosis; RTLE-no, right temporal lobe epilepsy without hippocampal sclerosis.

**Figure 4:**
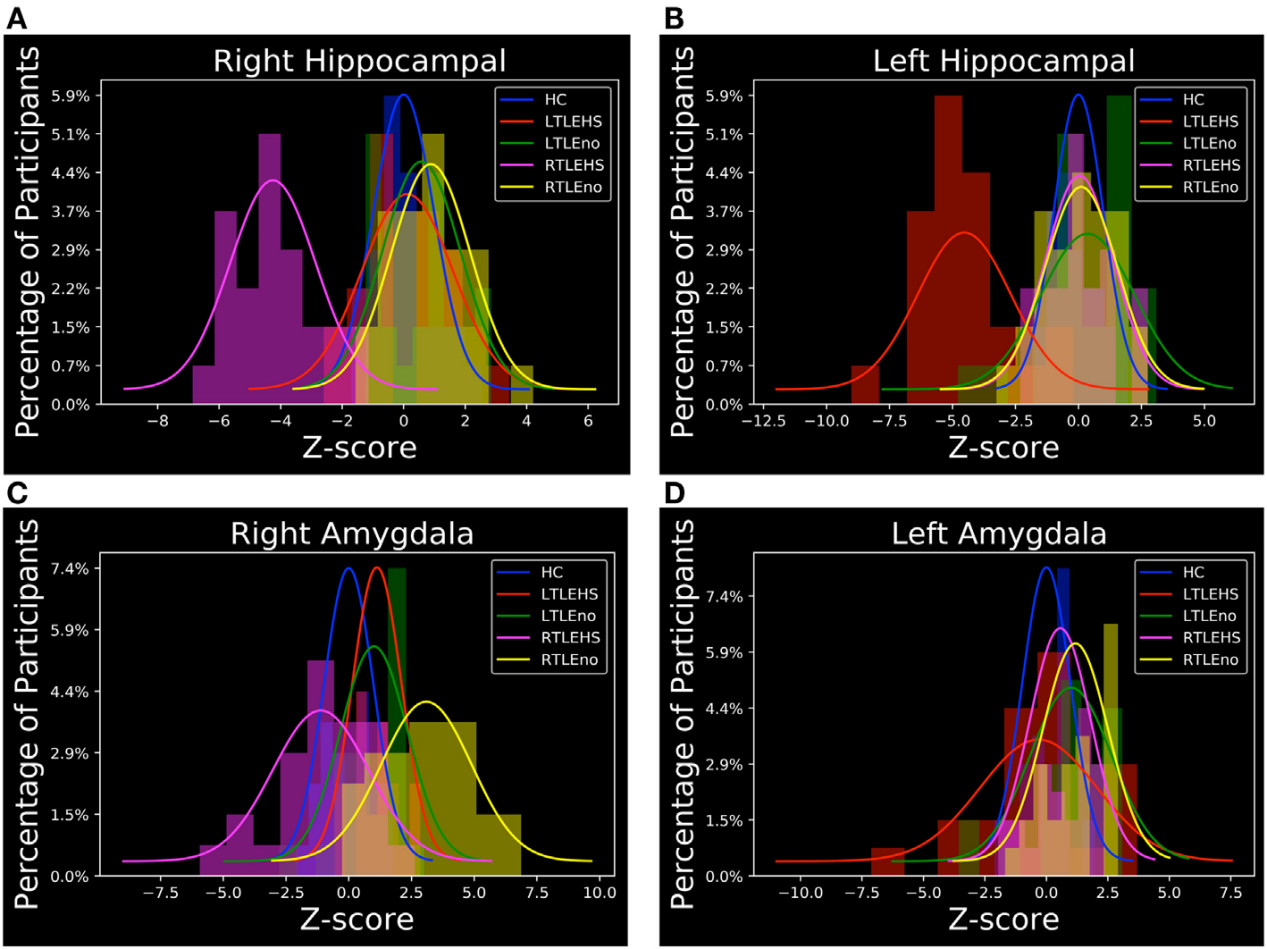
*Z* score distributions of hippocampal and amygdala volumes among the subject groups. **(A)** Right hippocampal, **(B)** left hippocampal, **(C)** right amygdala, and **(D)** left amygdala. HC, healthy control; LTLE-HS, left temporal lobe epilepsy with hippocampal sclerosis; LTLE-no, left temporal lobe epilepsy without hippocampal sclerosis; RTLE-HS, right temporal lobe epilepsy with hippocampal sclerosis; RTLE-no, right temporal lobe epilepsy without hippocampal sclerosis.

**Figure 5:**
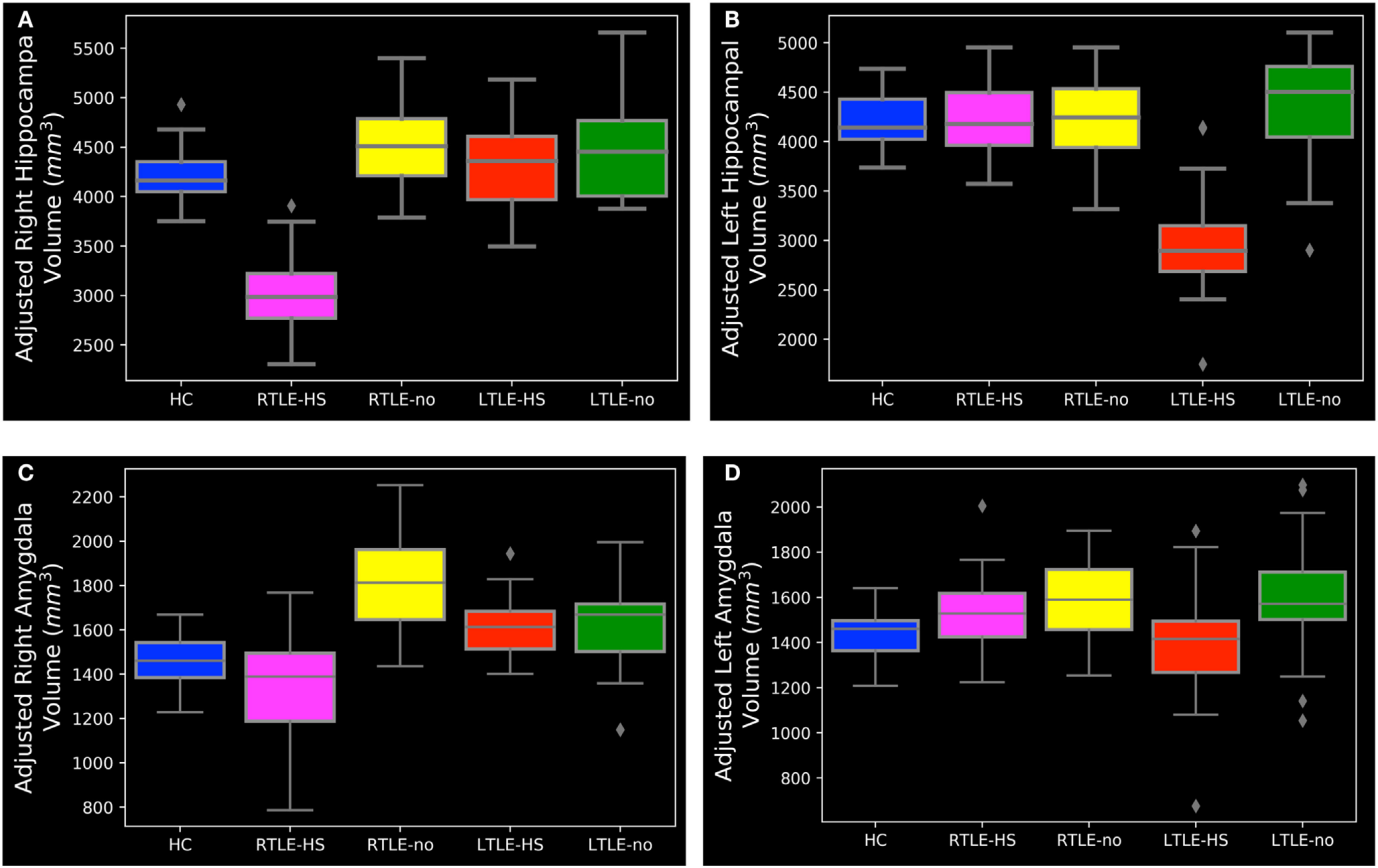
The range of hippocampal and amygdala volumes among the subject groups. **(A)** Right hippocampal, **(B)** left hippocampal, **(C)** right amygdala, and **(D)** left amygdala. HC, healthy control; LTLE-HS, left temporal lobe epilepsy with hippocampal sclerosis; LTLE-no, left temporal lobe epilepsy without hippocampal sclerosis; RTLE-HS, right temporal lobe epilepsy with hippocampal sclerosis; RTLE-no, right temporal lobe epilepsy without hippocampal sclerosis.

As described in Section “MRI Preprocessing,” we were interested in determining the hippocampal and amygdala volumes among the five subject groups. To this end, we performed individual segmentation using FreeSurfer to extract the volumes of the hippocampal and amygdala regions from all MRI scans. Figure 3 shows the association between the left and right hippocampal volumes as well as the amygdala volumes among the different groups. Figure 4 illustrates the distribution of *Z* scores of the hippocampal and amygdala volumes among the five subject groups. Regarding the right and left hippocampal volumes, 19.12% (24 RTLE-HS and 2 LTLE-HS patients) and 21.32% (22 LTLE-HS, 3 RTLE-HS, 2 RTLE-no, and 2 LTLE-no patients) of participants had a *Z* score lower than −2, respectively. With respect to the right and left amygdala volumes, 5.88% (seven LTLE-HS and one LTLE-no patients) and 4.41% (four RTLE-HS and two LTLE-no patients) of participants had a *Z* score lower than −2, respectively. Furthermore, 25% (21 RTLE-no, 8 LTLE-no, 4 LTLE-HS and 1 RTLE-HS patient) and 14.71% (9 RTLE-no, 6 LTLE-no, 3 LTLE-HS and 2 RTLE-HS patient) of participants had a *Z* score greater than 2 in the right and left amygdala volumes, respectively. Figure 5 shows the ranges of the left/right hippocampal and amygdala volumes among the five subjects groups. We conducted ANOVA to compare the left/right hippocampal and amygdala volumes. Regarding the hippocampal volumes obtained from the FreeSurfer analysis, the ANOVA test yielded *F* ratios of *F*(4,131) = 62.49 (*p* < 0.001) and *F*(4,131) = 46.96 (*p* < 0.001) related to the right and left hippocampal volumes, respectively, among the five groups. There were significant differences between the RTLE-HS patients and the HCs [mean difference (MD) = −1188.36, *p* < 0.001] and the LTLE-HS patients and the HCs (MD = −1234.76, *p* < 0.001) for the right and left hippocampal volumes, respectively. We also observed a significant interaction among the five groups related to the right and left amygdala volumes [*F*(4, 131) = 28.14, *p* < 0.001; and *F*(4, 131) = 4.87, *p* < 0.001, respectively].

As can be seen in Figures 3A and 5A,B, the RTLE-HS and LTLE-HS patients had significantly smaller right and left hippocampal volumes, respectively, than the RTLE-no, LTLE-no, and HC groups. In addition, the RTLE-no and LTLE-no patients had a pattern of right and left hippocampal volumes that was clearly similar to those of the HCs. Our statistical analysis of the hippocampal volumes obtained from FreeSurfer confirmed our VBM results, which showed no significant difference in hippocampal volume between the TLE-no patients and the HCs. As can be seen in Figures 3B and 5C, the RTLE patients without HS showed greater amygdala volume than the other groups. This finding concurs with our VBM results, which showed an increase in the GM in the amygdala region among the RTLE-no patients compared with the HCs.

### Regional Relationship between GM and WM Changes with Disease Duration

**Figure 6:**
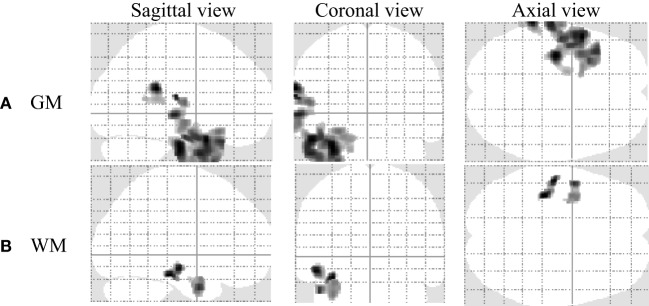
The result of a negative correlation of gray matter (GM) and white matter (WM) volumes with disease duration among left temporal lobe epilepsy patients with hippocampal sclerosis shown by multiple regression analysis using statistical parametric mapping software (uncorrected at *p* < 0.001 and an extent threshold *K* of 250). **(A)** GM and **(B)** WM.

As described in Section “Statistical Analysis,” we conducted multiple regression analysis to investigate the GM and WM changes with the duration of disease among our patient groups. We only observed negative significant GM and WM changes with disease duration in the LTLE-HS patient group. Figure 6 and Table 4 show the results of the negative relation between GM and WM volume changes with the disease duration through the multiple regression analysis in the LTLE-HS patient group. No significant positive interaction effects between the GM and WM volumes and disease duration were found.

**Table 4:** Clusters of negative gray matter and white matter alterations with duration disease among left temporal lobe epilepsy patients with hippocampal sclerosis shown by multiple regression analysis using statistical parametric mapping software.

Analysis	Location of peak voxels	Hemisphere	Cluster size (no. of voxels)	Talairach coordinates (*x*,*y*,*z*)	MNI coordinates (*x*,*y*,*z*)	*Z*-value (peak voxel)
(a) Gray matter	Inferior parietal lobule Parahippocampal PrimSensory	L L L	399 4,809 860	−64, −38, 22 −31, −18, −24 −63, −21, 15	−66, −39, 21 −33, −16, −32 −66, −21, 14	4.49 4.45 4.29

(b) White matter	Parahippocampal Fusiform	L L	280 412	−34, −28, −13 −46, −4, −25	−36, −27, −20 −48, 0, −32	4.25 3.93

*Anatomical regions were derived from the Talairach Client program*.

*L, heft hemisphere; R, right hemisphere; MNI, Montreal Neurological Institute (uncorrected at *p* < 0.001)*.

## Discussion

Several studies have applied neuroimaging techniques to the investigation of epilepsy under different contexts (24). Scanlon et al. (25) investigated the abnormality patterns in TLE-HS and TLE-no patients using tract-based spatial statistics and VBM analysis. They reported greater fractional anisotropy abnormalities in their TLE-HS patients compared with the TLE-no group and also described extensive extra-focal GM atrophy in both groups. Mueller et al. (7) examined the GM and WM abnormalities beyond the hippocampus between TLE-HS and TLE-no patients using an optimized VBM procedure. They reported GM and WM abnormalities in the ipsilateral limbic system, ipsi- and contralateral neocortices, thalamus, cerebellum, internal capsule, and brainstem regions for TLE-HS patients compared with HCs. They did not observe any differences in GM or WM abnormalities between TLE-no patients and HCs or between TLE-HS and TLE-no patients.

Ahmadi et al. (8) examined the side matters in TLE patients using diffusion tensor imaging analysis. They reported a widespread reduction in fiber tract fractional anisotropy in the TLE patients and more diffuse changes in left TLE patients compared with right TLE patients. McMillan et al. (26) used the VBM technique to investigate GM alterations in TLE. They reported GM abnormalities in ipsilateral hippocampal and ipsilateral thalamic regions. They also stated that chronic TLE is associated not only with abnormalities in GM, but also with concomitant abnormalities in cerebral WM regions. Sone et al. (6) investigated the brain abnormalities in TLE patients suffering from AE in a comparison with TLE-HS patients and HCs. Their findings revealed a mesial temporal decline in TLE-HS patients and an increase in GM in the amygdala among TLE-AE patients. They also observed a significant reduction in WM in the ipsilateral temporal lobe of TLE-HS patients compared with HCs.

In the present study, we conducted a whole-brain VBM analysis to investigate the GM and WM volume abnormalities among five groups of participants (i.e., HC, RTLE-HS, RTLE-no, LTLE-HS, and RTLE-no). To evaluate the VBM results, we also performed a FreeSurfer volumetric analysis of the subjects’ hippocampal and amygdala regions. The VBM analysis revealed ipsilateral mesiotemporal volume reductions in both the GM and WM in the TLE-HS patients. This finding is in line with those of other studies reporting a volume reduction that included extra-hippocampal areas in TLE-HS patients (6, 11). Some studies stated extra-temporal widespread atrophy as well as mesial temporal atrophy in TLE patients (11, 27), whereas our present group comparisons with the latest software (SPM12) and a rigorous statistical analysis revealed that only mesiotemporal atrophy would remain, although the degree and extent of atrophy would be expected to differ among individuals.

We also identified a slight amygdala GM swelling in the RTLE-no patients. The amygdala, as the center of emotional behavior, plays an important role in epilepsy patients (28). There have been several reports on TLE with ipsilateral AE (29–31). Such patients might have been present in our TLE-no group. Additionally, according to a recent machine-learning MRI study (32), this kind of medial temporal GM increase has been systematically classified as a subtype of TLE. However, there is another discussion about the longitudinal amygdala volume changes (33), suggesting that AE can be partly explained by an inflammatory process or seizure activity.

We observed no other abnormalities in the TLE-no patients. There is much to be elucidated beyond morphology in TLE-no, in addition to its potential heterogeneity. A variety of pathological findings in the enlarged amygdala have been reported, including focal cortical dysplasia, low-grade glioma, clustering hypertrophic neurons, and vacuolation (34, 35). In our previous study (36), we conducted the VBM analysis using two versions of the widely applied SPM toolbox (CAT12 and VBM8^5^) for detecting brain morphological abnormalities in TLE patients with and without HS. We observed different patterns of GM and WM abnormalities in TLE between the VBM8 and CAT12 programs. In the current study, we performed a volumetric analysis using FreeSurfer to confirm the results obtained from VBM analysis using CAT12.

Our volumetric analysis demonstrated that about 90% of TLE patients with HS had a significant ipsilateral hippocampal atrophy (i.e., 24 of 26 RTLE-HS and 22 of 25 LTLE-HS) compared to HCs. This finding is relatively consistent with those of other studies (37) which stated a significant ipsilateral hippocampal atrophy in 91% of the 45 TLE patients without foreign tissue lesion, in which most cases showed HS in the pathology. Besides, we observed that about 20% of TLE patients with HS had a significant ipsilateral amygdala atrophy (i.e., 4 of 26 RTLE-HS, and 7 of 25 LTLE-HS) compared to HCs, whereas some studies (37) reported an ipsilateral amygdala atrophy in 91% of the TLE who without foreign tissue lesion which most of them had HS in pathology. The reasons for this discrepancy are not clear, but may be due to the selection of patients, different segmentation methods or imaging qualities. It is worth noting that compared to hippocampal atrophy, amygdala volume decline is still controversial in TLE (11).

Furthermore, we explored the regions of GM and WM alterations with disease duration in our patient groups and observed significant negative GM and WM changes with disease duration in the LTLE-HS patients. Our present finding is in contrast to some studies (38) which reported no changes in GM or WM with the duration of epilepsy. Although there is still controversy about the progression of atrophy in TLE, a recent meta-analysis revealed evidence of progressive GM atrophy, especially on the ipsilateral side (39). However, there are still questions about side-specific differences (i.e., left or right focus of TLE) and WM findings. Our results may partly explain these differences, suggesting significant correlations with disease duration in both GM and WM atrophy only in LTLE-HS. Although our regression results were not very robust statistically, left TLE might be more predisposed to atrophy and WM could also be affected by disease progression in more limited areas.

Our present study has some limitations. First, we compared five different patient groups and the size of each group was thus relatively small. Given the rigorous statistical analysis, our results may have lower power to detect any other potential abnormality, although the significance obtained would be reliable. Second, the effects of drugs, disease duration, and the heterogeneity of TLE-no as potential confounders were not examined.

## Conclusion

We performed a whole-brain VBM analysis to explore the GM and WM volume abnormalities among five groups of subjects. We also performed a statistical analysis of the amygdala and hippocampal volumes extracted from FreeSurfer for comparison with our VBM findings. We observed ipsilateral mesiotemporal volume reductions in both the GM and WM in TLE-HS patients and slight amygdala swelling in RTLE-no patients through VBM analysis. The volumetric analysis through FreeSurfer authenticated the VBM findings of significant atrophy in the left and right hippocampal in LTLE-HS and RTLE-HS patients as well as a slight swelling in the amygdala volume in the RTLE-no patients, respectively, compared with the HCs. We also observed a significant negative GM and WM alterations with disease duration only in the LTLE-HS patients. Identification of the differences in GM and WM abnormalities between epilepsy patients and healthy subjects may help researchers to better decipher an individual’s response to drug therapy.

## Ethics Statement

All participants gave written informed consent. The study was approved by the Institutional Review Board at the National Center of Neurology and Psychiatry Hospital.

## Author Contributions

IB, DS, and FF generated the research idea, literature search, and concept. DS acquired the data. NM performed the volumetric segmentation. IB and DS analyzed the data. IB and FF drafted the work. IB wrote the manuscript. HM participated in the design of the study and supervised the analysis. All authors read and approved the final manuscript.

## Conflict of Interest Statement

None of the authors has any conflict interest. We confirm that we have read the Journal’s position on issues involved in ethical publication and affirm that this report is consistent with those guidelines.

## Footnotes

^1^www.mccauslandcenter.sc.edu/crnl/mricron/.

^2^http://dbm.neuro.uni-jena.de/cat.

^3^http://www.fil.ion.ucl.ac.uk/spm/software/spm12/.

^4^https://surfer.nmr.mgh.harvard.edu/.

^5^http://www.neuro.uni-jena.de/vbm/.
